# Targeting a novel tamoxifen-using pathway to preserve ovarian reserve in rats with experimental chemotherapy-induced ovarian failure

**DOI:** 10.1186/s40360-026-01103-5

**Published:** 2026-03-11

**Authors:** Amira S. Ahmed, Mahmoud S. Sabra, Asmaa Youssef A. Abbas, Asmaa A. Kamal, Zainab S. Abdelqader

**Affiliations:** 1 Histology Department, Faculty of Medicine, Assiut University, Assiut, 71526 Egypt; 2 Department of Pharmacology, Faculty of Veterinary Medicine, Assiut University, Assiut, 71526 Egypt; 3 Department of Medical Biochemistry, Faculty of Medicine, Assiut University, Assiut, 71526 Egypt

**Keywords:** Cyclophosphamide, Oxidative stress, Premature ovarian insufficiency, Anti-inflammatory cytokines, Mitochondrial fission

## Abstract

**Background:**

Due to the increased incidence of cancer in young women, chemotherapy-induced gonad toxicity, such as that caused by cyclophosphamide (CP), is now the main cause of premature ovarian insufficiency (POI).

**Aims:**

The current work investigates how CP damages the rat ovary and whether tamoxifen (TAM) medication and pretreatment can reduce CP by restoring antioxidant and mitochondrial balance.

**Methods:**

Forty adult female albino rats were randomly allocated into four groups. The control group and CP-induced POI group were used. TAM pretreatment for four weeks prior to CP injection in the third group. A single dosage of TAM was administered concurrently with CP in the fourth group.

**Results:**

In the group receiving CP, the following observations were observed: a reduction in the ovarian index; a decline in the numbers of primordial and primary follicles; an increase in atretic follicles; a decrease in mRNA expression of insulin-like growth factor 1 (IGF-1) and its receptor (IGF-1R); a significant reduction in anti-Müllerian hormone (AMH); an increase in serum levels of dynamin-related protein (DRP-1); a decrease in serum levels of interleukin 10 (IL-10), nitric oxide (NO), and mitofusin 1 (MFN-1); a damaged ovarian tissues that evaluated histopathologically; an decrease in the number of primordial and primary follicles; an increase in the number of atretic follicles; zona pellucida’s integrity disruption; an decrease in ovarian weights; and an increase in collagen fiber area percentages; a decrease in heme oxygenase-1 (HO-1) immunoreactivity; and an increase in the expression of immunopositive cells for cluster of differentiation 86 (CD86) compared to controls. In contrast, both treatment and pretreatment with TAM improved these effects. With concern to pretreatment with TAM that showed a superior improvement in increasing the number of primordial and primary follicles, improving cortical follicles, enhancing zona pellucida’s integrity and increasing in HO-1immunoreactivity combined with limited regions of collagen fibers.

**Conclusion:**

One possible explanation for the protective effects of TAM on ovarian tissue is that it increases IGF-1 mRNA expression, improves mitochondrial dynamic balance, and activates the IL-10/HO-1 pathway, all of which work together to decrease oxidative stress and increase ovarian tissue survival. Based on our research, it appears that a potentially effective regimen for POI caused by CP is pretreatment with TAM. This could lead to a higher percentage of POI disease remission.

## Introduction

One common chemotherapeutic drug used to treat erratic cancer types is CP. Despite its adverse effects, it is a very effective drug when used either alone or in combination with another chemotherapeutic [1]. Chemotherapy-induced gonadotoxicity is now the primary cause of ovarian failure due to the rising frequency of cancer in young women. CP negatively impacts female reproductive organs, particularly the ovary [2].

One member of the family of selective estrogen receptor modulators that prevent estrogen from attaching to the receptor is TAM. It is recognized to exert diverse effects on organs, including beneficial estrogenic actions in bones and liver, as well as favorable estrogen antagonistic activity in breast cancer tissue [3]. Since the first clinical trials in the early 1970s, TAM has been used extensively to treat estrogen receptor-positive breast cancer. TAM’s anticancer effects are mainly due to the way it modulates gene expression through competitive blockage of the estrogen receptor, which stops breast cancer cells from proliferating and increases their death [4]. According to one study, for instance, TAM causes cell death via altering the transcriptional control of the expression of the apoptosis-related Bcl-2 family members [5]. However, mounting data suggests that TAM, at relatively high concentrations, has additional estrogen receptor-independent anticancer actions in various cancer cell types [6, 7].

Chemotherapeutic agents like CP can induce excessive reactive oxygen species, impair DNA structure and function in ovarian granulosa cells, and initiate apoptosis in oocytes, leading to irreversible premature ovarian insufficiency (POI) [8]. Elevated reactive oxygen species can influence signaling pathways, including phosphatidylinositol 3-kinase/protein kinase B, mitogen-activated protein kinase, and Kelch-like ECH-associated protein [9]. Increased Bcl-2-associated X protein expression reduces mitochondrial membrane potential, leading to cytochrome C release, caspase family activation, apoptosis, follicular atresia, and accelerated progression of POI [10].

Tamoxifen has been documented to possess a protective effect against both radiotherapy-induced ovarian follicular depletion [11] and chemotherapy-induced POI [12]. According to a prior study, TAM had a radioprotective effect and preserved ovarian reserve and fertility by raising local IGF-1 levels and AMH levels and preventing oxidative stress-induced apoptosis [11]. The ovarian transcriptomes and proteomes showed consistent modifications after TAM treatment, suggesting that anti-apoptotic pathway activation may be involved in TAM’s protective effects. The genes that showed differential expression were associated with DNA repair pathways, cell adhesion-related activities, and extracellular matrix remodeling as potential players in these processes [13]. Furthermore, a new study found that TAM protects the ovaries through molecular mechanisms that involve lncRNA-dependent regulation of important signaling pathways that suppress apoptosis, regulate cell adhesion, inhibit follicular transition, and slow ovarian aging [14]. Moreover, the molecular mechanisms of TAM activity in tumor-bearing rats’ ovaries were also found to be related to basic metabolic processes, signaling pathways that prevent the activation of primordial and primary follicles, and steroid hormone synthesis [13]. Since there are currently no established treatments that can restore a woman’s ovaries’ fertility or even normal functionality following chemotherapeutic treatment [15, 16], our findings may provide a novel approach to prevent chemotherapy-induced primary ovarian insufficiency utilizing TAM.

Heme oxygenase-1 (HO-1, heat shock protein 32) is an enzyme that can be activated by stress. Multiple gynecological malignancies, including endometrial, cervical, and ovarian cancers, have recently been found to have significant HO-1 expression, which raises the possibility that this oncogene is involved in cell proliferation, metastasis, immunological modulation, and angiogenesis [17]. Additionally, the role of antioxidant and anti-inflammatory markers, such as HO-1 and IL-10, in preventing ovarian failure was documented [18]. Nevertheless, TAM therapy increased the amounts of HO-1 protein in mice’s adipose tissue [19].

Recent research has looked at the connection between autoantibodies in POI and peripheral blood regulatory T cells. In POI, they found that the peripheral blood had more CD4 and CD69 activated T cells and fewer effector regulatory T cells, indicating that POI is an autoimmune illness [20]. Additionally, antigen-presenting cells express the molecule CD86, which is associated with immunological responses [21], including autoimmune processes that might result in POI.

To the best of our knowledge, however, no study has examined how pretreatment with TAM affects HO-1 and CD86 and how it relates to anti-inflammatory and antioxidant indicators as well as mitochondrial fission and fusion dynamics. Thus, we set out to test a new hypothesis about the role of mitochondrial and inflammatory pathways in POI in rats that had been pretreated with TAM and then exposed to CP-induced POI. Moreover, the current investigation examines the mechanism by which CP damages the rat ovaries and whether TAM treatment or pretreatment can reduce CP by reestablishing the balance of antioxidants, inflammatory factors, and mitochondrial dynamics.

## Materials and methods

### Animals and experimental design

The study comprised forty female albino rats, housed in stainless steel cages with adequate ventilation and temperature control. The rats were kept in a light-regulated environment (12-hour light/dark cycle), with unrestricted access to water and provided daily with a standard pellet diet.

Cyclophosphamide dose (150 mg/kg) was chosen for this study due to its toxicity demonstrated in the ovaries of rats and mice [15, 22]. The UK-based pharmaceutical firm AstraZeneca supplied TAM citrate (Nolvadex 10 mg) tablets.

**Fig. 1:**
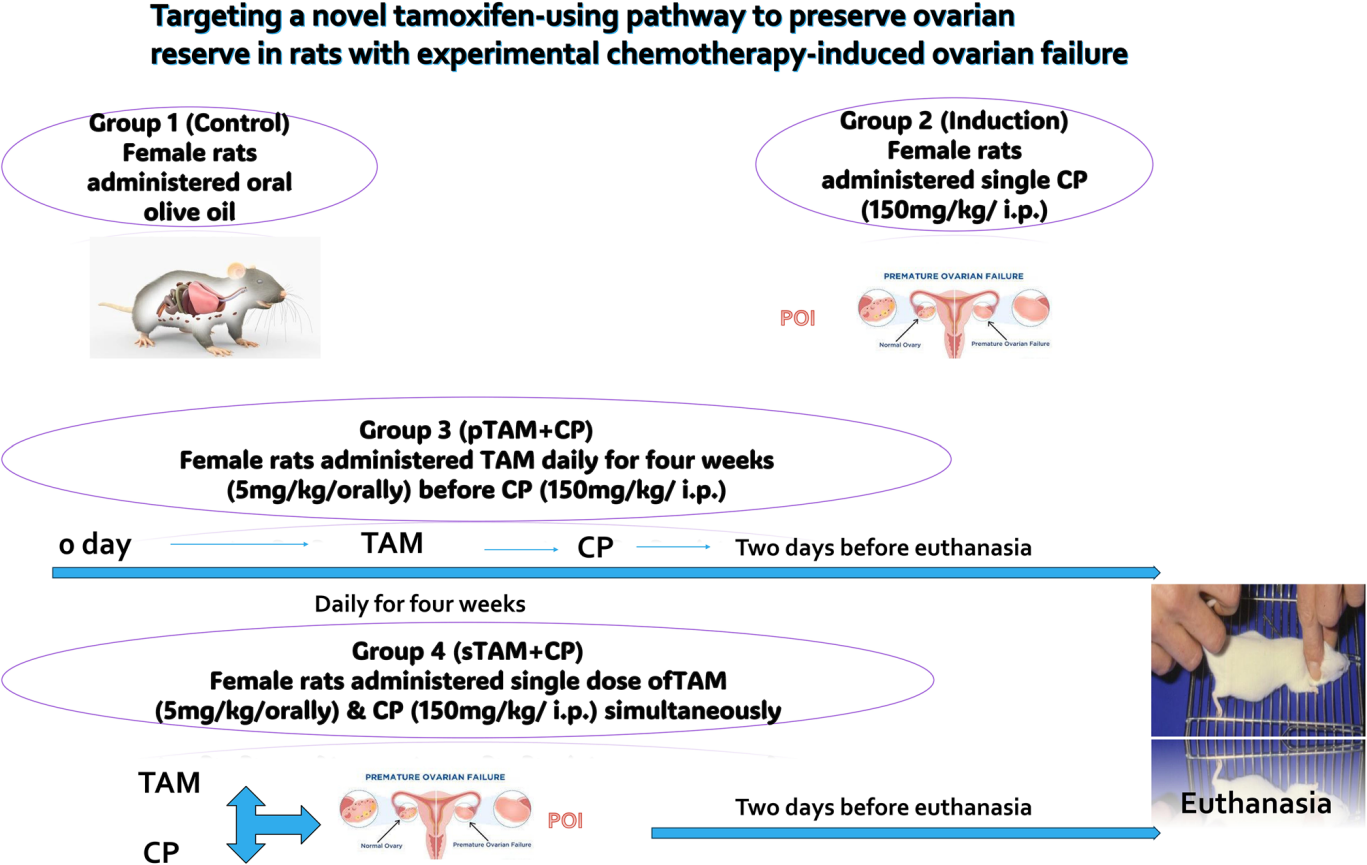
Design of the experimental groups. Graphical depiction of the experimental rat cohorts utilized for investigating both tamoxifen treatment and pretreatment in conjunction with cyclophosphamide (CP)-induced premature ovarian insufficiency (POI). The rats were categorized into four groups: control, POI-induced, TAM pretreatment for four weeks before CP-induced POI (pTAM + CP), and single dose TAM treatment in CP-induced POI (sTAM + CP)

The rats (150–200 g, 3 months old) were randomly assigned into four groups (*n* = 10 rats /group). Group I (control group) rats received olive oil; group II (CP group) rats received a single intraperitoneal (ip) injection of CP (150 mg/kg); group III (pTAM + CP) rats, similar to group II, received CP, but in addition, for 4 weeks preceding the CP injection, they also received daily TAM (5 mg/kg, orally dissolved in olive oil) [23] before CP induction of POI; and finally, group IV (sTAM + CP) rats received a single dose of TAM (5 mg/kg, orally) simultaneously with the CP injection (150 mg/kg, ip) (Fig. 1).

### Collection of ovaries and serum

Blood samples were drawn from the retro-orbital plexus using a glass capillary tube under light anesthesia. All animals were later euthanized with 5% isoflurane [24] followed by neck dislocation. Serum was separated by centrifugation (700×g, 20 min.) and stored at -80 °C until determination of AMH, NO, IL-10, MFN-1, and DRP-1 concentrations. All ovaries were collected and washed with ice-cold phosphate-buffered saline. One ovary was kept for 24 h in 4% paraformaldehyde for immunohistochemical and histological examination. The second ovary, designated for biochemical and molecular analysis, was kept at -80 °C after being snap-frozen in liquid nitrogen.

### Enzyme linked immunoassays

#### Measurement of AMH serum level

AMH serum concentration was measured by enzyme-linked immunoassay (ELISA) Kit (ELK Biotechnology, catalog no. ELK0211) according to the manufacturer’s recommendations.

#### Measurement of IL-10 and NO serum concentrations

Serum levels of IL-10 and NO were measured by ELISA Kits (Thermo Fisher Scientific Inc., USA, catalog no. ERA23RB and SunLong Biotech Co., LTD, Hangzhou City, Zhejiang Province, China, catalog no. SL0531Ra, respectively) according to the manufacturer’s recommendations.

#### Determination of mitochondrial dynamic indicators

An ELISA Kit (catalog no. SL1810Ra) for MFN-1 serum analysis and an ELISA Kit (catalog no. SL1868Ra) for DRP-1 serum analysis were purchased from SunLong Biotech Co., LTD and were used in accordance with the instructions provided by the manufacturer.

### Real-time quantitative polymerase chain reaction (qPCR)

Total RNA was extracted from ovarian tissue utilizing the RNeasy Mini Kit (Catalog no. 74104, Qiagen, Germany). The purity and concentration of the RNA fraction were assessed using a Nanodrop^®^ (Epoch Microplate Spectrophotometer; Biotech). Complementary DNA (cDNA) was synthesized employing the Thermo Scientific High-Capacity cDNA Reverse Transcription kit (catalog no. 4368813). qPCR was performed using the Maxima SYBR Green qPCR master mix (2×) kit (Thermo Scientific, catalog. no. #K0251). The Applied Biosystems 7500 Fast Real-time PCR machine (Applied Biosystems) was used for RT‐qPCR with beta actin as the internal control. The PCR cycling conditions after optimization were as follows: initial denaturation step at 95° C for 7 min (1 cycle), followed by 40 cycles of 95 °C for 15 s and then 60 °C for 55 s. The relative transcription levels of mRNA were calculated using the equation of fold change 2 − ΔΔCT method. Table 1 gives a list of primers for IGF-1, IGF-1R, and β- actin used in the investigation.

**Table 1:** Lists the primers for qPCR. All primers were synthesized by thermo fisher Scientific, USA

Primer	Forward	Reverse	Accession number
IGF − 1	CAAAATGAGCGCACCTCCAATA	TTGAGGGAAATGCCCATCTCTG	NM_178866.4
IGF-1R	AAACGCTGACCTCTGTTAC CTCTC	GCGGATGAAGCCTGATGGAC	NM_001414181.1
B-actin	GATTACTGCCCTGGCTCCTAGC	CTCCTGCTTGCTGATCCACATC	NM_031144.3

Insulin-like growth factor 1 (IGF-1) and its receptor (IGF-1R)

### Histological examination of ovarian tissues

Before the experiment, the estrous cycle was checked to confirm that all rats were on the second day [25]. Only animals with regular estrous cycles were tested. Vaginal smears were obtained everyday (8–10 am) for seven days before the trial. Rats go through proestrus (P), estrus (E), metestrus (M), and diestrus (D) in 4–5 days. The Histology Department, Faculty of Medicine, Assiut University, examined the smears under a light microscope.

After the experiment, all ovaries were stored in 10% formalin and processed as formalin-fixed paraffin-embedded tissue slices for histopathological and immunohistochemical tests. To assess histological architecture, paraffin-embedded tissue slices were cut to 5–7 μm thickness using a microtome and stained with H&E stain to examine ovarian structure under light microscopy. Masson’s trichrome stain showed ovarian tissue fibrosis, and periodic acid-Schiff [19] assessed zona pellucida integrity. Five slides from each group were randomly viewed and photographed under a light microscope. Three histopathologists were blinded to the groups being researched. Five fields from each image were randomly examined. ImageJ quantified fibrosis area [3, 26].

### Immunohistochemistry (IHC)

Paraffin-embedded ovarian tissue sections were mounted on poly-L-lysine slides, deparaffinized in xylene, then rehydrated in descending alcohol grades. In a 10-um citrate buffer for 10 min, tissue was heated to retrieve antigen. The sections were then treated with avidin-biotin-peroxidase complex and primary antibodies for HO-1 (ab13248) and CD86 (Thermo Scientific, MA5-13324). The sections were dehydrated, washed, and counterstained with Meyer’s hematoxylin for light microscopy. 3,3’-diaminobenzidine-hydrogen peroxide visualized the response [27–29]. HO-1 and CD86 negative controls from the kidney and colon sections were used to validate the IHC interpretation [30, 31]. Additionally, morphometric analysis quantified HO-1 and CD86 immunopositive cells.

### Morphometric analysis of HO-1 and CD86 immunopositive cells and follicle number

The ovarian tissues were rinsed with ice-cold phosphate-buffered saline and weighed. The high-resolution digital camera on a computer-linked microscope photographed paraffin-embedded ovarian tissue slices stained with HO-1 and CD86 after tissue processing and staining. Image processing software (IMAGE-J, NIH OCI, USA) quantified HO-1 and CD86 immunopositive cells, the percentage area of collagen fibers in Masson trichrome stained sections, and the count of primordial, primary, and atretic follicles to assess ovarian follicle reserve. Measuring each group’s specimens in 10 randomly selected non-overlapping fields at 400 magnifications [22, 32]. Ovarian reserve requires primordial and primary follicles, hence only these were selected. Reproduction relies on the non-growing follicular reserve in early development [33]. Morphology defined follicular type [34].

### Statistical analysis

Parametric statistical analysis was performed as a result of the Shapiro-Wilks normality test. Data were expressed as mean ± SD. Statistical analysis was conducted using GraphPad Prism software (Version 8). One-way analysis of variance (ANOVA) followed by the Tukey post hoc test was employed to assess differences among the experimental groups. A P value of < 0.05 was deemed statistically significant.

## Results

### Mitochondrial homeostasis/inflammatory markers and antioxidants

In the CP-treated group, serum concentrations of NO and IL-10 considerably decreased (*p* < 0.0001) compared to the control group; however, TAM in both the pTAM + CP and sTAM + CP groups dramatically restored (*p* < 0.0001) their levels.

**Fig. 2:**
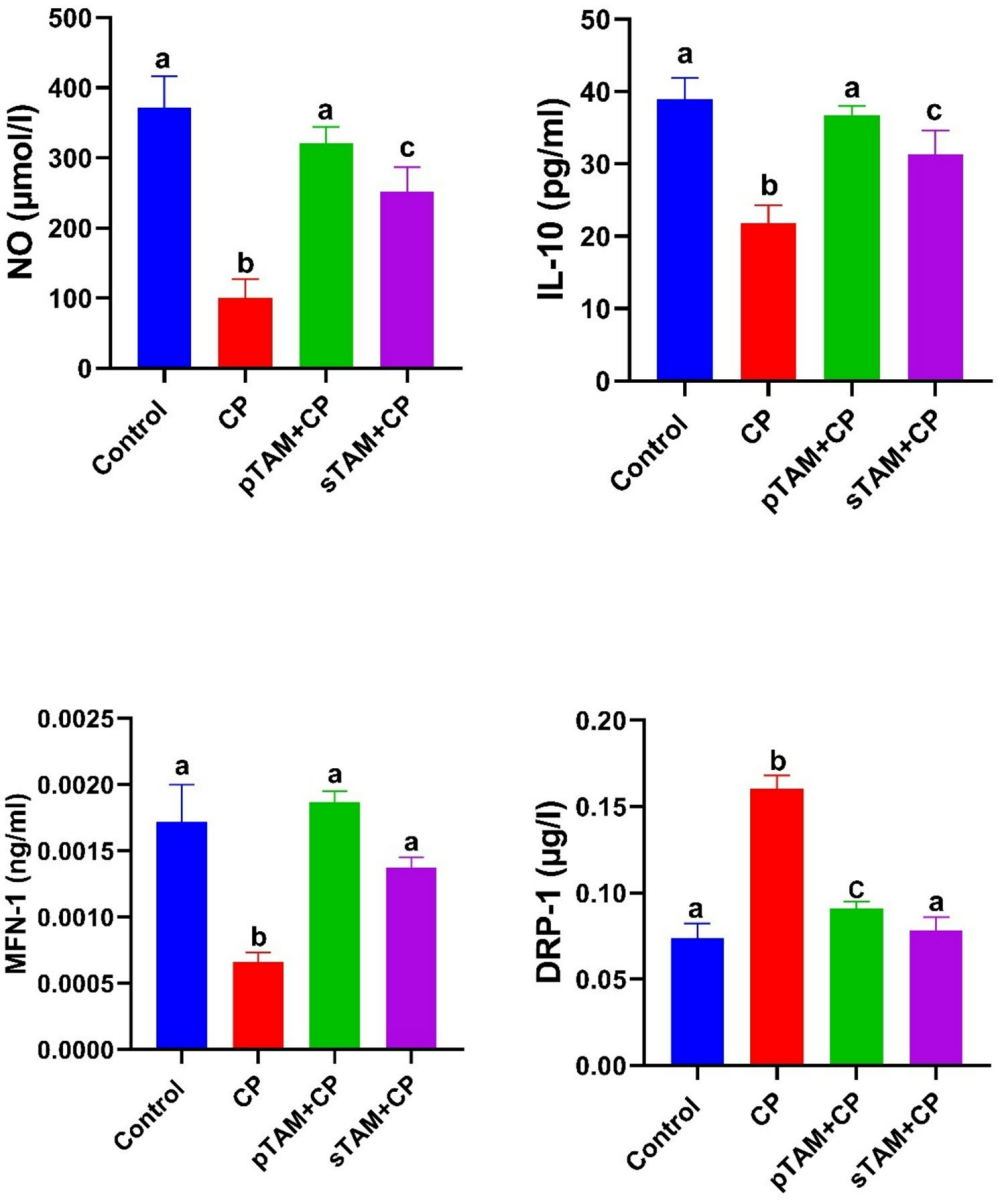
Impact of tamoxifen administration on serum concentrations of nitric oxide (NO), interleukin-10 (IL-10), mitofusin 1 (MFN-1), and dynamin-related protein (DRP-1) in rats with cyclophosphamide-induced ovarian failure. CP (Cyclophosphamide-treated), pTAM + CP (Tamoxifen-pretreated for four weeks) and sTAM + CP (Tamoxifen-treated simultaneously with CP). Different letters indicate significant differences among treatments (*p* < 0.05) (One-way ANOVA, followed by Tukey post hoc test)

Similarly, serum MFN-1 concentration was significantly lower (*p* < 0.001) in the CP-treated group than in the control group. Nonetheless, MFN-1 levels were significantly elevated in the TAM of both the pTAM + CP (*p* < 0.001) and sTAM + CP (*p* < 0.01) groups. On the contrary, serum DRP-1 level was significantly increased (*p* < 0.0001) by CP treatment. TAM, in both TAM groups, restored the control levels of DRP-1 (*p* < 0.0001) (Fig. 2).

### Serum AMH and the number of primordial, primary and atretic follicles

**Fig. 3:**
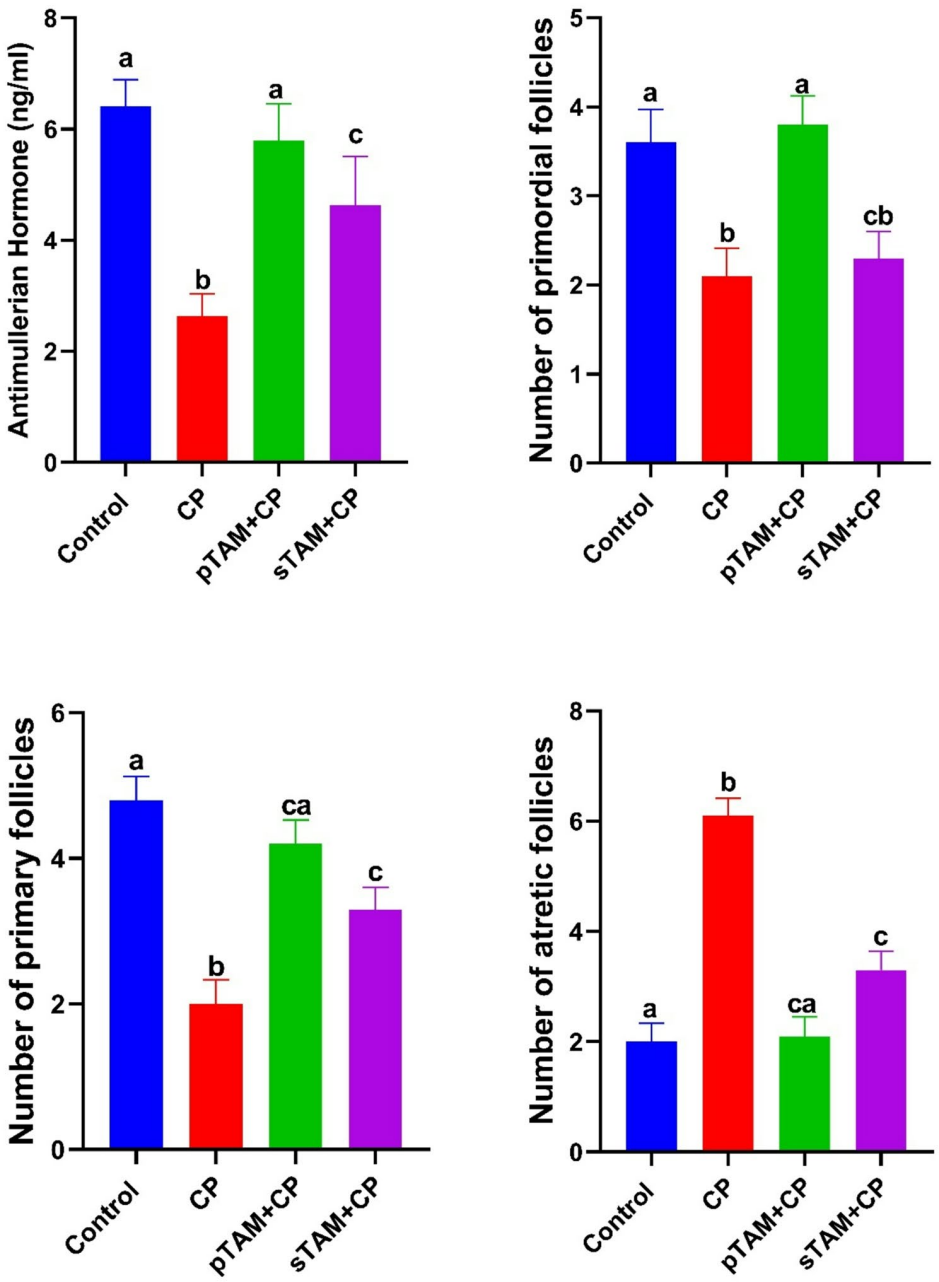
Effects of tamoxifen treatment on serum levels of anti-müllerian hormone (AMH) and quantitative assessment of primary, primordial, and atretic follicle counts in rats with cyclophosphamide-induced ovarian insufficiency. CP (Cyclophosphamide-treated), pTAM + CP (Tamoxifen-pretreated for four weeks) and sTAM + CP (Tamoxifen-treated simultaneously with CP). Different letters indicate significant differences among treatments (*p* < 0.05) (One-way ANOVA, followed by Tukey post hoc test)

In the CP-treated group, serum AMH level exhibited a substantial reduction (*p* < 0.0001) compared to the control group. The AMH level was significantly increased (*p* < 0.0001) by TAM of both groups to its control level (Fig. 3).

The number of primordial and primary follicles was markedly decreased (*p* < 0.05, *p* < 0.0001, respectively) by CP. Compared to the CP-treated group, pTAM + CP considerably elevated (*p* < 0.001, *p* < 0.0001) the counts of primordial and primary follicles, whereas the sTAM + CP group significantly enhanced (*p* < 0.05) the number of primary follicles without affecting primordial follicles. This effect was more pronounced in the pTAM + CP group. In turn, the number of atretic follicles was significantly higher (*p* < 0.0001) in the CP-treated group than in the control group. Again, TAM of both groups restored (*p* < 0.0001) the number of atretic follicles almost to its control values (Fig. 3).

### Ovarian histology

**Fig. 4:**
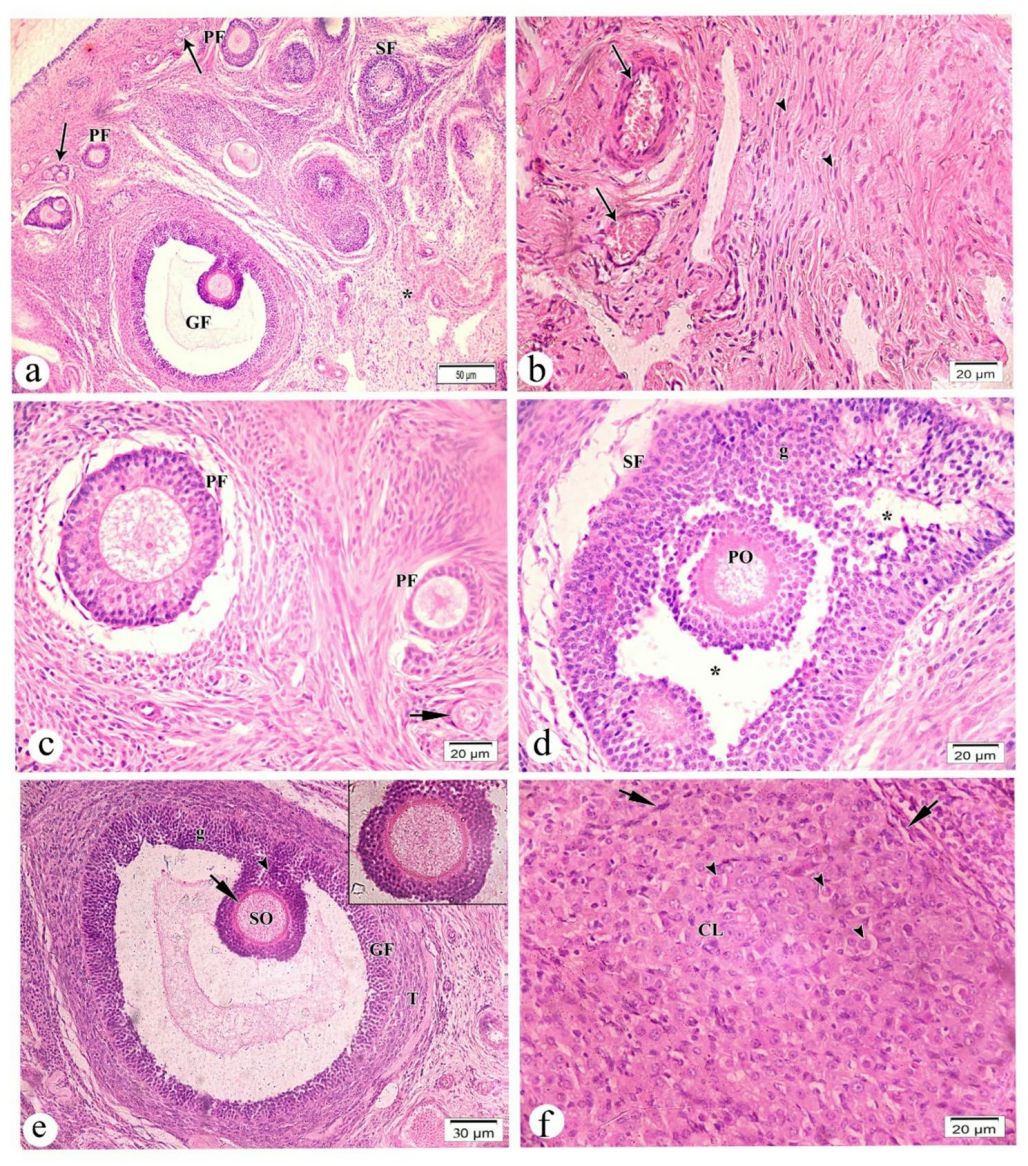
Light photomicrographs of control group (group I) rat’s ovarian tissue sections stained with hematoxylin and eosin (H&E). **a**. low magnification showing the classical structure of ovarian cortex that is occupied by multiple follicles included primordial (arrows), primary (PF), secondary (SF) and Graffian follicle (GF). Notice part of ovarian medulla (*) is present. **b**. high magnification of ovarian medulla showing blood vessels with mild vascular congestion (arrows) surrounded by scattered smooth muscles (arrow heads). **c**. high magnification of ovarian cortical follicles showing primordial follicle (short arrow), unilamminar and multilaminar types of (PF). **d**. high magnification of secondary follicle (SF) showing a central primary oocyte (PO) surrounded by multiple layers of granulosa cells (g), separated by multiple antral spaces (*). **e**. high magnification of mature graffin follicle (GF) consisting of secondary oocyte (SO) surrounded by thick membrane of zona pellicida (short arrow), cumulus oophorus (arrow heads), granulose cells (g), and differentiated theca cells (T). **f**. high magnification of corpus luteum cellular structure (CL) consisting of central vacuolated granulosa lutein cells (arrow heads) and peripherally located smaller and darker theca lutein cells (short arrows). Magnification (**a**) x 100 (**b**, **c**, **d**, **f**) x400, (**e**) x 200 and inset x400

Control rats had primordial, primary, secondary (SF), and Graafian (GF) follicles in their ovarian cortex (Fig. 4a). Desmosomes connect a primary oocyte to a single layer of squamous follicular (pregranulosa) cells from the neighboring ovarian connective tissue (stroma) in primordial follicles. The ovarian medulla had mild vascular congestion and scattered smooth muscle fibers (Fig. 4b). Ovarian cortical follicles had primordial, unilaminar, and multilaminar primary follicles. Multiplelaminar or late primary follicles have a stratified follicular epithelium with gap connections between the granulosa cells, while unilaminar ones have an oocyte in a simple cuboidal epithelium (Fig. 4c). Antral gaps and multiple layers of granulosa cells surrounded the main oocyte in the SF (Fig. 4d). The mature Graafian follicle had a secondary oocyte, cumulus oophorus, granulosa cells, and differentiated theca cells (Fig. 4e). The corpus luteum had core vacuolated granulosa lutein cells, big polygonal cells with light eosinophilic cytoplasm and conspicuous spherical nuclei, and peripheral theca lutein cells, smaller, darker cells.

**Fig. 5:**
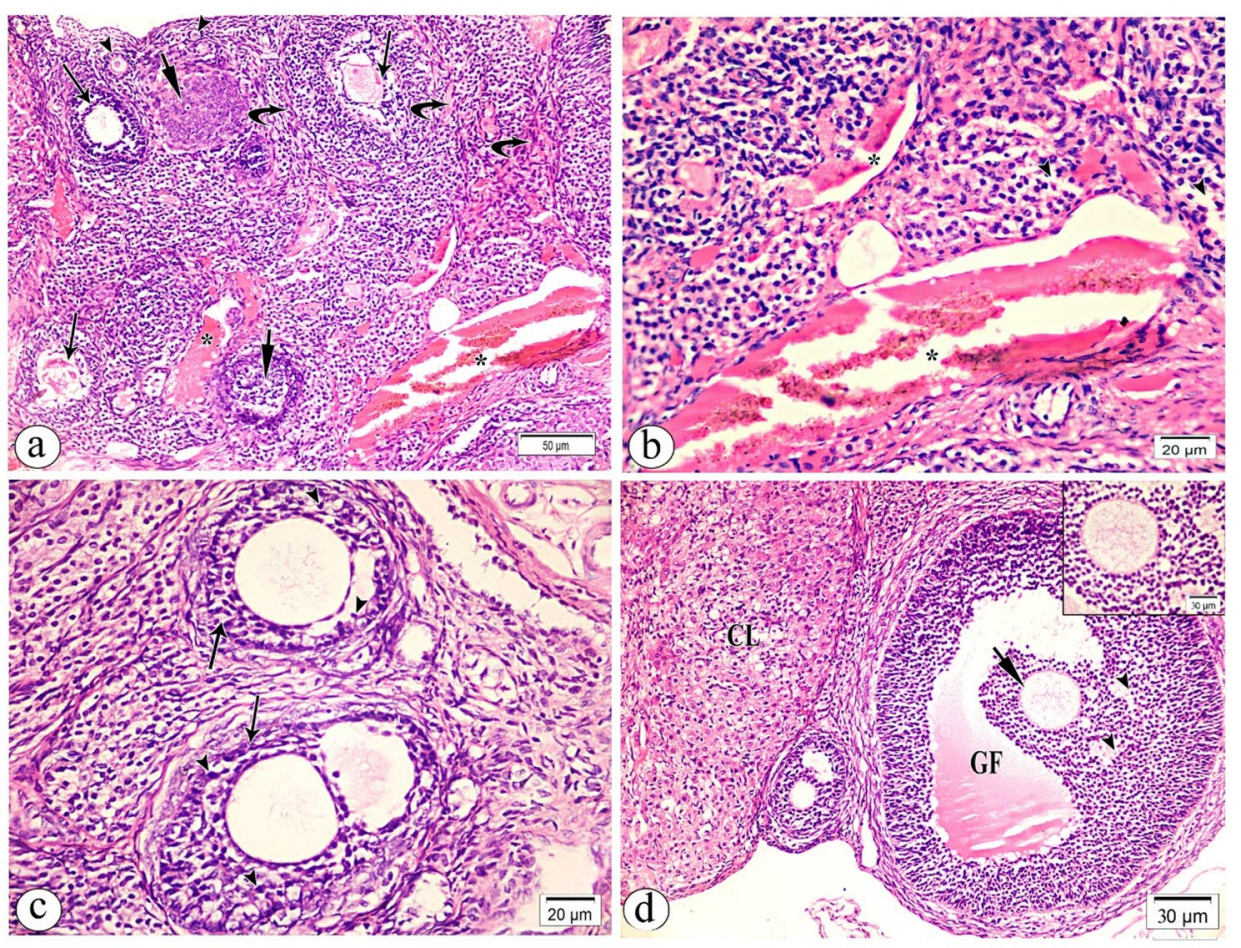
Light photomicrographs of cyclophosphamide treated group (group II) of ovarian tissue sections stained with hematoxylin and eosin (H&E). (**a**) low magnification showing the ovarian cortex occupied by multiple degenerated (arrows) and atretic follicles (short arrows) in addition to few intact small cortical growing follicles (arrow heads). Part of ovarian medulla with marked thickened blood vessels (*), and excess fibrous tissue deposition (curved arrows) in the ovarian stroma are noticed. (**b**) high magnification of ovarian medulla showing marked thickened and hyalinized blood vessels (*) separated by rarified tissue (arrow heads). (**c**) high magnification of degenerated follicles (arrows) showing degenerated granulose cells with dense nuclei (arrow heads). In addition to ill -defined oocytes and zona pellucida membrane within the degenerated follicles are present. (**d**) high magnification of degenerated graffin follicle (GF) showing thinning of zona pellucida membrane (short arrow) and spaced rarified granulosa cells (arrow heads). Notice part of corpus luteum CL and inset of graffin follicle (GF) with dense nuclei of many degenerated granulosa cells (arrow heads). are present. Magnification (**a**) x 100, (**b**, **c**) x400 (**d**) x 200 and inset x400

In the CP-treated group, ovarian cortex slices showed many degraded and atretic follicles and a few undamaged small cortical developing follicles. A part of the ovarian medulla had dilated, thickened-wall hyalinized blood vessels and fibrous tissue in the stroma (Fig. 5a). The ovarian medulla had rare tissue and hypertrophied hyalinized blood vessels (Fig. 5b). The degenerated follicles had degraded granulosa cells with dense nuclei, poorly defined oocytes, and zona pellucida membranes (Fig. 5c). The degenerated GF had a thin zona pellucida membrane and scattered sparse granulosa cells. Many destroyed granulosa cells had dense nuclei in the corpus luteum and GF (Fig. 5d).

**Fig. 6:**
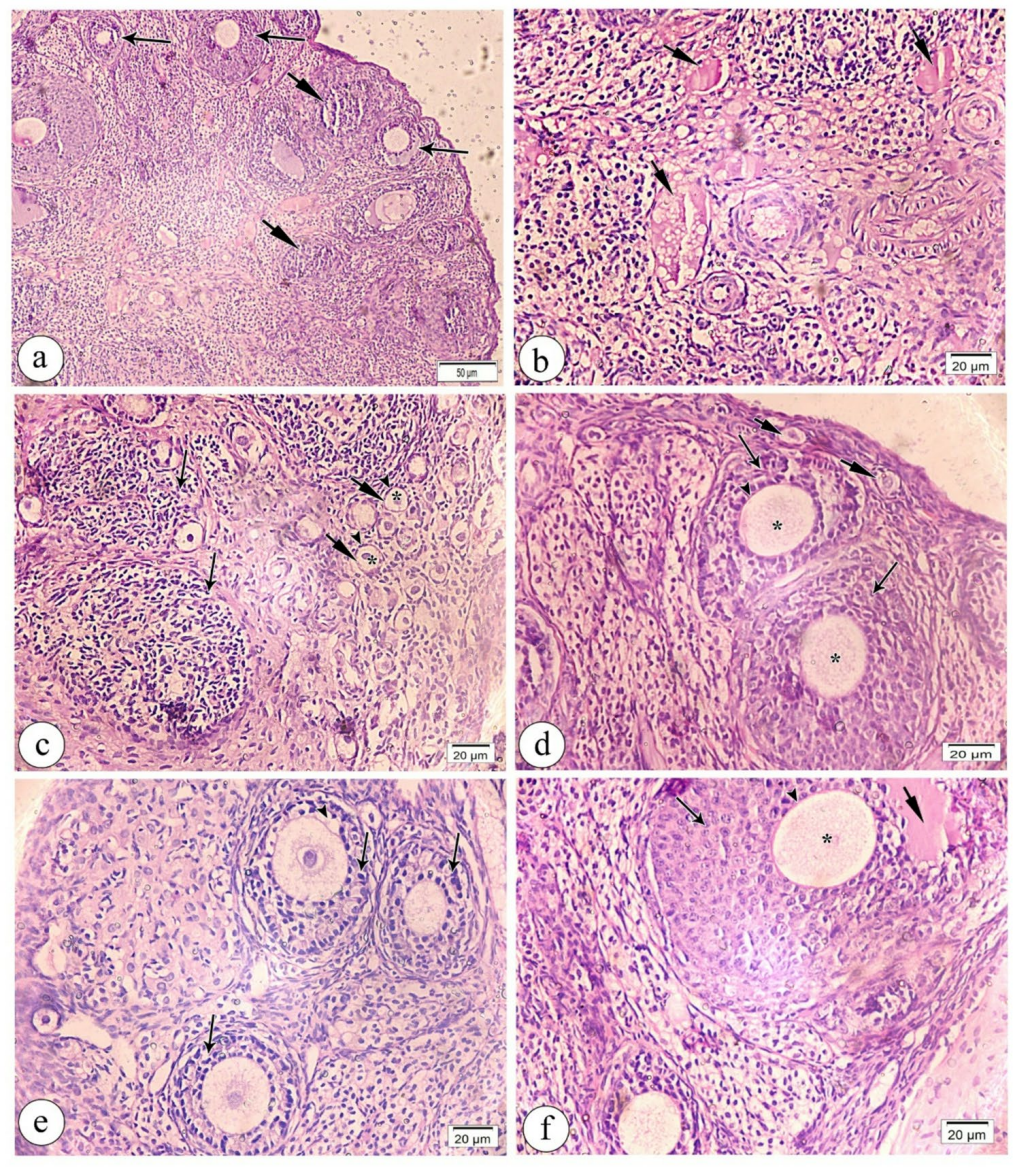
Light photomicrographs of TAM pretreated group (pTAM + CP) of ovarian tissue sections stained with hematoxylin and eosin (H&E). **a**. low magnification showing multiple cortical growing follicles (arrows) with few degenerated follicles (short arrows).**b**. high magnification of ovarian cortex showing multiple primordial follicles (short arrows) with central primary oocytes (*) surrounded by single layer of stromal cells (arrow heads) adjacent to few degenerated follicles (arrows).**c**. high magnification revealing two adjacent multilaminar primary follicles (arrows) with primary oocytes (*) that surrounded by zona pellucida membrane (arrow heads), in addition to cortical primordial follicles (short arrows). **d**. high magnification showing three adjacent multilaminar primary follicles (arrows) with degeneration of some surrounded granulosa cells arrow heads). **e**. high magnification of large secondary follicle (arrow) reaveling antral space (short arrow) within the granulosa cells, primary oocytes (*) that surrounded by zona pellucida membrane (arrow heads). **f**. high magnification of ovarian medulla showing less dilated hyalinized blood vessels (short arrows). Magnification (**a**) x 100, (**b**-**f**) x400

Ovarian sections from the pTAM + CP group showed many growing cortical follicles and few damaged ones (Fig. 6a). Several deteriorated follicles were near primordial follicles with core primary oocytes surrounded by a single layer of stromal cells in the ovarian cortex (Fig. 6b). Figure 6c shows cortical primordial follicles and two multilaminar primary follicles with primary oocytes enclosed in zona pellucida membranes. In three contiguous multilaminar primordial follicles, some granulosa cells degenerated (Fig. 6d). We also saw large secondary follicles with antral gaps inside the granulosa cells and zona pellucida membrane-encased primary oocytes (Fig. 6e). The ovarian medulla had less blood channel dilatation and hyalinization (Fig. 6f).

**Fig. 7:**
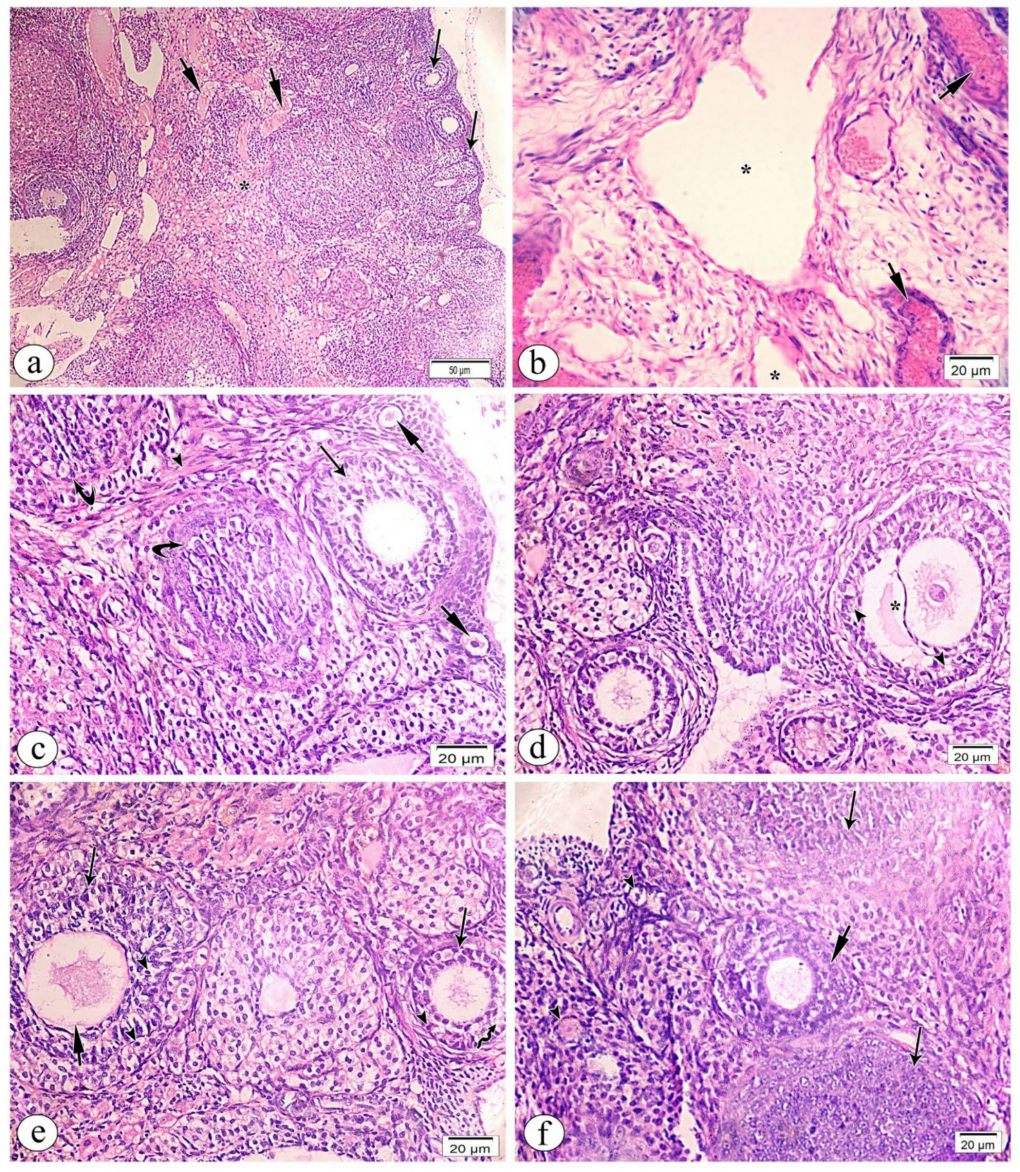
Light photomicrographs of sTAM + CP group of ovarian tissue sections stained with hematoxylin and eosin (H&E). **a**. low magnification showing different stages of ovarian follicles (arrows) additionally to part of medulla (*) with congested blood vessels (short arrows) is observed. **b**. high magnification of ovarian medulla showing wide spacing within rarified tissue (*) with congested vessels (short arrows). **c**. high magnification of cortical primary follicles (arrow) adjacent to some primordial follicles (short arrows) in addition to some atretic follicles (curved arrows) separated by excess fibrous tissue (arrow head). **d**. high magnification of degenerated follicle showing ill-defined zona pellucida membrane with marked separation (*) within rarified degenerated granulosa cells (arrow heads). **e**. high magnification of two multilaminar primary follicles revealing rarified zona pellucida membrane (arrows) surrounded by many spaced rarified granulosa cells (short arrows) some of them with dense nuclei (arrow heads). **f**. high magnification of two large atretic follicles (arrows) that adjacent to growing primary follicle (short arrow) and few primordial follicles (arrow heads). Magnification (**a**) x 100, (**b**-**f**) x400

In the sTAM + CP group, ovarian follicles were at varied stages, and the medulla had congested blood arteries (Fig. 7a). The ovarian medulla had thicker hyalinized arteries and considerable spacing inside attenuated tissue (Fig. 7b). An abundance of fibrous tissue separated cortical primary and atretic follicles from primordial follicles (Fig. 7c). Indistinct zona pellucida membrane with substantial separation amid rarefied degraded granulosa cells characterized the damaged follicle (Fig. 7d). The two multilaminar primary follicles featured a rarefied zona pellucida membrane with sparsely dispersed rarefied granulosa cells, some with dense nuclei (Fig. 7e). Two large atretic follicles were near developing main and primordial follicles (Fig. 7f).

### Zona pellucida’s integrity, ovarian weights, and collagen fiber area percentages

**Fig. 8:**
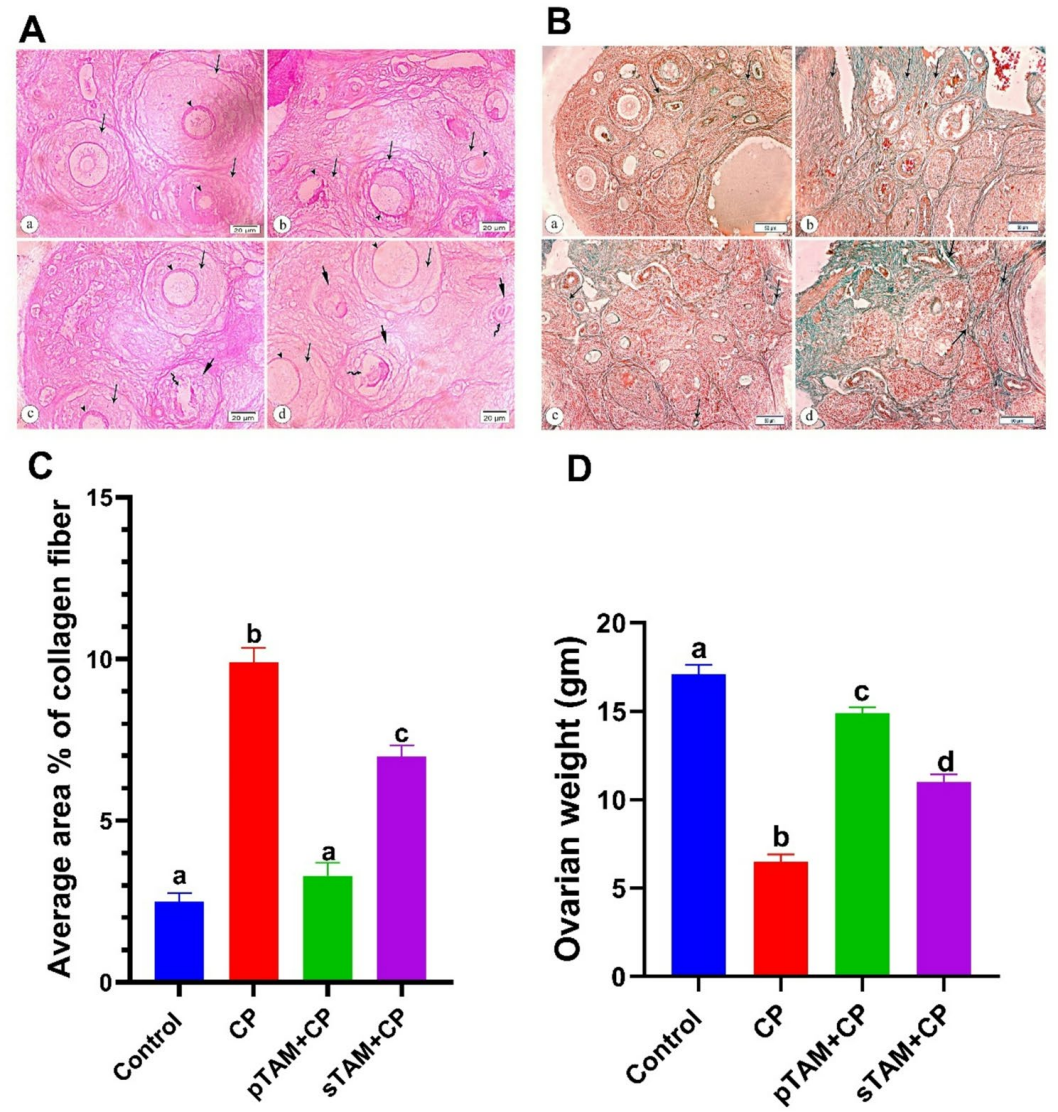
**A**) Light photomicrographs of rat’s ovarian tissue sections stained with periodic acid Schiff x400. **a**. Control group showing positive PAS reaction of intact thick surrounded zona pellucida (arrow heads) of adjacent growing follicles (arrows). **b**. CP-induced POI group showing positive PAS reaction of irregular and interrupted thin zona pellucida (arrow heads) of many degenerated follicles (arrows). **c**. pTAM + CP group show positive PAS reaction on intact thick surrounded zona pellucida (arrow heads) of adjacent growing follicles (arrows). Notice degenerated follicle (short arrow) with damaged zona pellucida, and its oocyte (wavy arrow) is present. **d**. sTAM + CP group showing positive PAS reaction of intact zona pellucida (arrow heads) of growing follicles (arrows) that are adjacent to some degenerated follicles (short arrows) with damaged zona pellucida (wavy arrow). **B**) Light photomicrographs of rat’s ovarian tissue sections stained with Masson trichrome stain at x100 magnification. **a**. Control group showing a few green colored collagen fibers surrounding and between the follicles and around blood vessels (arrows). **b**. CP-induced POI groupshowing marked increase in the amount of collagen fibers within the ovarian stromal tissue (arrows) compared to group I. **c**. pTAM + CP group exhibiting small area of collagen fibers (arrows) surrounding the different follicles. **d**. sTAM + CP group revealing an increase in the amount of collagen fibers (arrows) surround the follicles. **C**) Average area % of collagen fibers in all studied groups. **D**) Ovarian weight changes in all studied groups. CP (Cyclophosphamide-treated), pTAM + CP (Tamoxifen-pretreated for four weeks) and sTAM + CP (Tamoxifen-treated simultaneously with CP). Different letters indicate significant differences among treatments (*p* < 0.05) (One-way ANOVA, followed by Tukey post hoc test)

In the intact thick zona pellucida enclosing developing follicles, the control group had a positive PAS reaction. With an irregular and fragmented thin zona pellucida in many damaged follicles, the CP-treated group had a faint positive PAS result. On intact, thick zona pellucida surrounding nearby follicles, pTAM + CP had a good PAS reaction. A deteriorated follicle with a weakened zona pellucida and oocyte revealed. Near some damaged follicles with compromised zona pellucida, the sTAM + CP group showed a modest positive PAS reaction (Fig. 8A).

Using Masson Trichrome stain, the control group showed green collagen fibers around follicles and blood vessels. CP-treated ovarian stromal tissue has more collagen fibers than group I. A restricted area of collagen fibers surrounded the follicles in the pTAM + CP group. The sTAM + CP increased follicular collagen fibers (Fig. 8B).

Average collagen fiber area percentage was considerably (*p* < 0.0001) higher in the CP-treated group compared to the control group. Collagen fiber % reduced (*p* < 0.0001) after TAM treatments in both pTAM + CP and sTAM + CP groups (Fig. 8C).

In the CP-treated group, ovarian weights significantly decreased (*p* < 0.0001) compared to the control group. In both pTAM + CP and sTAM + CP groups, post-treatment ovarian weights decreased (*p* < 0.0001) in TAM (Fig. 8D).

### Ovarian IHC of heme oxygenase-1 and CD86 cells

**Fig. 9:**
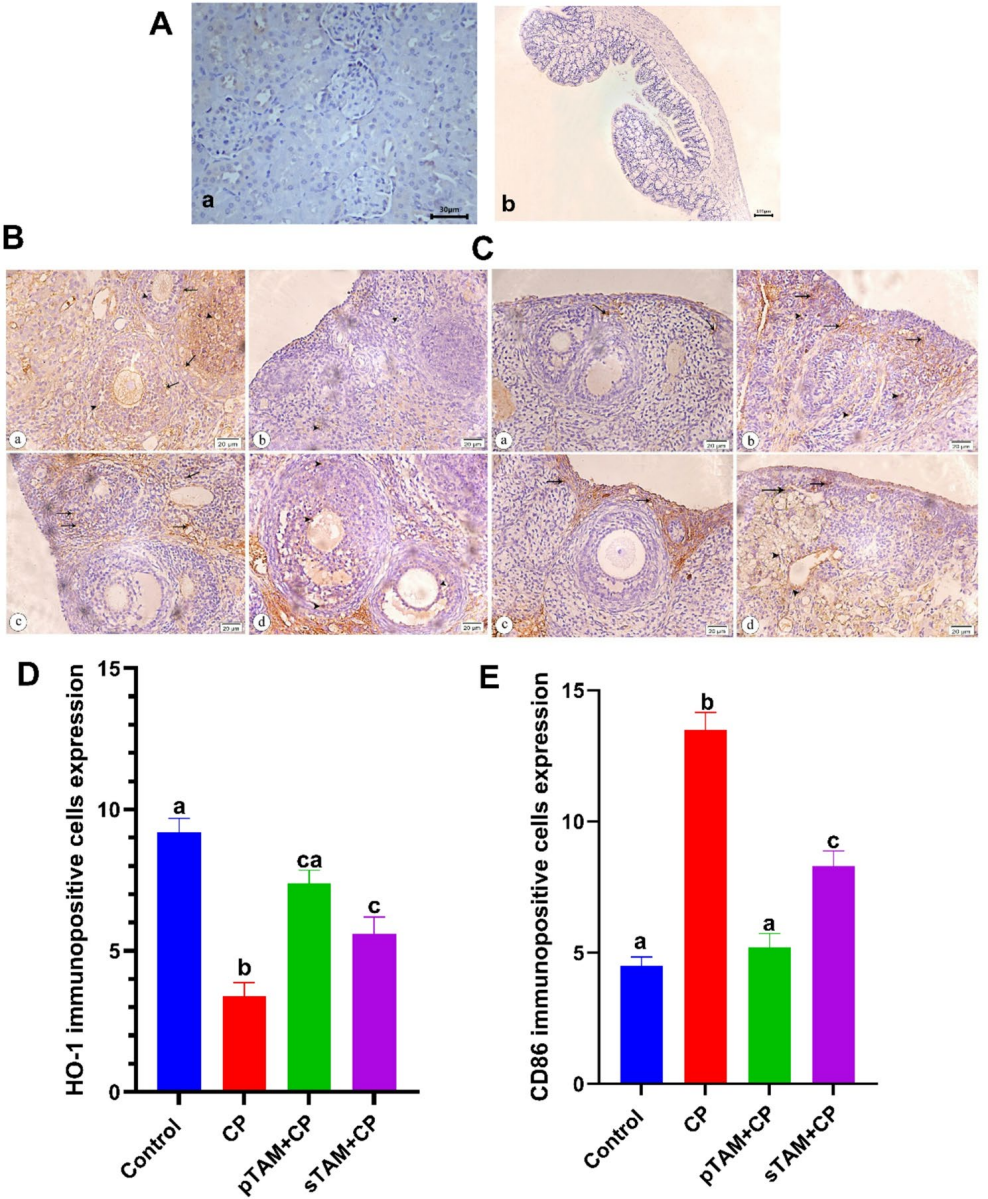
**A**) Light photomicrographs of kidney and colon sections immunostained with heme oxygenase 1 (HO-1) and cluster of differentiation 86 (CD86), respectively (**a**) Kidney section showing negative immunostaining reaction for HO-1 at x400 magnification. (**b**) Colon sectionshowing negative immunostaining reaction for CD86 at x100 magnification. **B**) Light photomicrographs of rat’s ovarian tissue sections stained with HO-1 immunostaining at x400 magnification. **a**. Control group showing strong positive immunostaining reaction within the cytoplasm of many granulosa (arrow heads) and letual cells (arrows) of the growing follicles. **b**. CP-induced POI group showing faint positive brown immunostaining area of few granulosa cells (arrow heads). **c**. pTAM + CP group exhibiting a marked increase in positive immunostained cells (arrows) compared to group II. **d**. sTAM + CP group revealing moderate increase in the positive immunostained granulosa cells (arrow heads) of the follicles. **C**) Light photomicrographs of rat’s ovarian tissue sections stained with CD86 immunostaining at x400 magnification. **a**. Control groupshowing few positive CD86 immunostained cells especially in subcapsular region (arrows). **b**. CP-induced POI group showing marked increase in the positive immunostained macrophages in subcapsular (arrows) and in the stroma (arrow heads) between the follicles compares to group I. **c**. pTAM + CP group exhibiting many positive immunostained cells in subcapsular region (arrows). **d**. sTAM + CP group revealing many positive immunostained macrophages in subcapsular region (arrows) and surrounded the blood vessels (arrow heads). **D**) HO-1 quantitative analysis in all studied groups. **E**) CD86 quantitative analysis in all groups studied. CP (Cyclophosphamide-treated), pTAM + CP (Tamoxifen-pretreated for four weeks) and sTAM + CP(Tamoxifen-treated simultaneously with CP). Different letters indicate significant differences among treatments (*p* < 0.05) (One-way ANOVA, followed by Tukey post hoc test)

Negative kidney and colon controls with low HO-1 and CD86 protein expression. Both parameters were negligible in sections (Fig. 9A). The control group showed a strong positive HO-1 antibody immunological marker reactivity, which was seen in the cytoplasm of numerous granulosa lutein cells in growing follicles. Few CP-treated granulosa cells showed modest brown immunostaining. Compared to CP, pTAM + CP had more positively immunostained cells. The sTAM + CP had moderately more positively immunostained granulosa cells in follicles (Fig. 9B). A quantitative analysis of HO-1 immunopositive cell expression showed a significant increase (*p* < 0.0001) in the pTAM + CP group compared to the CP-treated group (Fig. 9D).

In the subcapsular area, the control group had few CD86-positive cells. Contrary to control group, CP-treated group had more positively immunostained macrophages in the subcapsular region and stroma between follicles. Many subcapsular cells were immunostained with pTAM + CP. Many positively immunostained macrophages surrounded the blood arteries and subcapsular portions of the sTAM + CP (Fig. 9C). TAM therapy significantly reduced CD86 immunopositive cell expression (*p* < 0.0001) in both the pTAM + CP and sTAM + CP groups compared to the CP-treated group (Fig. 9E).

### IGF-1 and IGF-1R gene expression

**Fig. 10:**
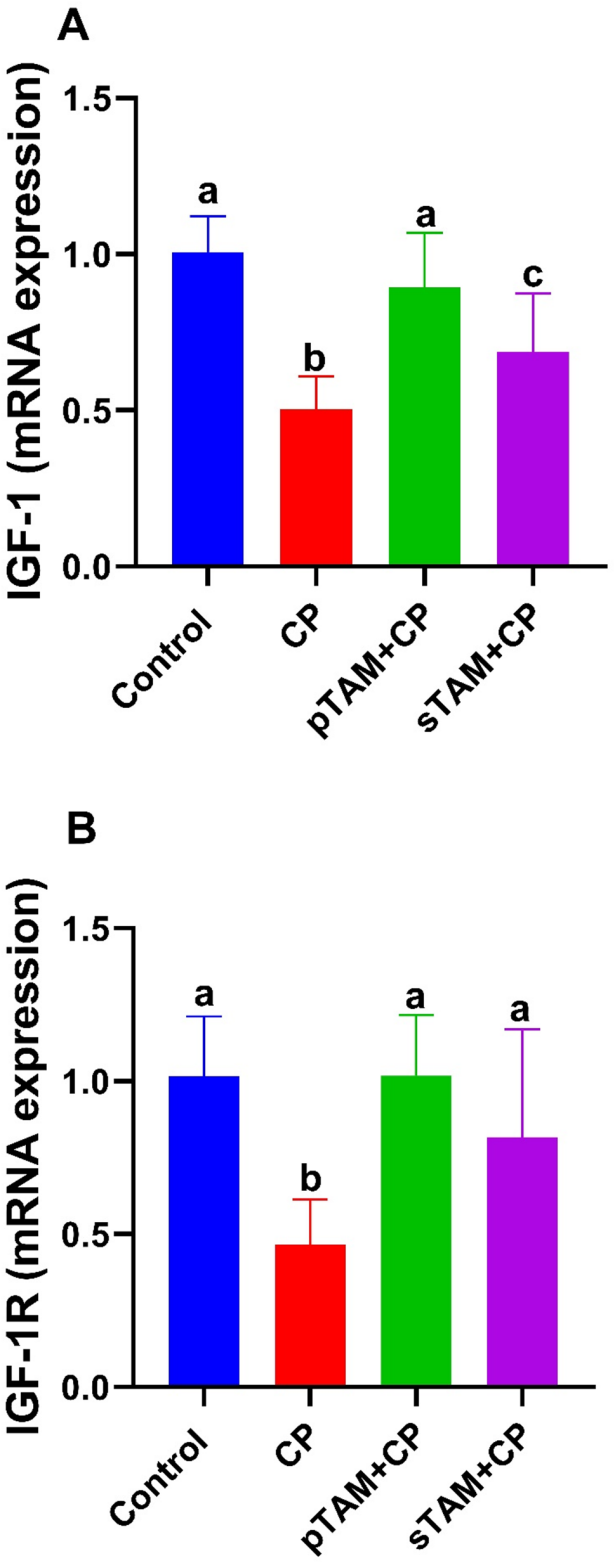
Real-time quantitative polymerase chain reaction for insulin-like growth factor 1 (IGF-1) and its receptor (IGF-1R) across all examined groups. CP (Cyclophosphamide-treated), pTAM + CP (Tamoxifen-pretreated for four weeks) and sTAM + CP (Tamoxifen-treated simultaneously with CP). Different letters indicate significant differences among treatments (*p* < 0.05) (One-way ANOVA, followed by Tukey post hoc test)

Cyclophosphamide decreased (*p* < 0.0001) mRNA expression of IGF-1 and IGF-1R. However, TAM restored the expression of both genes (*p* < 0.0001) to their control values (Fig. 10).

## Discussion

About 1% of women under 40 and 0.1% of women under 30 suffer from POI, which is defined as the loss of typical ovarian function before the age of 40. Ovarian sex hormone deficiency and low ovarian reserve cause premature menopause and a rapid decline in ovarian function in POI. Biochemically, it manifests as hypoestrogenic and hypergonadotropic symptoms, which can lead to infertility in some women. The process of controlling and maintaining the quality and quantity of ovarian follicles requires additional investigation, and there are still many unanswered questions about the causes driving ovarian dysfunction, even though reproductive endocrinology has made great strides recently [35].

In the fields of medicine and dermatology, immunosuppressant medications are prescribed for a wide variety of severe and long-lasting conditions. Immunosuppressants include various medications, including CP and methotrexate [36]. As an alkylating agent, CP can damage DNA molecules, which can result in cell death, mutagenesis, or carcinogenesis [37]. CP is toxic to both dividing and non-dividing cells because it destroys DNA. The ovarian reserve of oocytes/ovarian follicles is reduced because CP dose-dependently kills both active and latent ovarian follicles [38]. Oocyte numbers dramatically decreased, leading to POI, with higher doses and longer durations [39].

The inflammatory response elicited by chemotherapeutic agents has been documented in prior studies, including inflammatory cell infiltrations and heightened expression of proinflammatory cytokines, which correlates with impaired ovarian function and subsequent damage to adjacent tissue. This damage may endure post-chemotherapy, leading to a condition of sustained immune activation and chronic inflammation [40]. Furthermore, mitochondria have a crucial role in steroidogenesis, which happens in the cumulus granulosa and theca cells, in addition to their roles in oxidative phosphorylation, thermogenesis, apoptosis, and reactive oxygen species equilibrium [41].

POI may involve inflammatory and immunological molecules as HO-1 and IL-10 [42]. Both in vitro and in vivo animal models showed that NO and HO-1 are essential for steroid production, ovarian follicle growth, and oocyte maturation [43]. IGF-1 and IGF-1R enhance granulosa cell differentiation and proliferation [44]. It inhibits apoptosis, prevents follicular atresia, promotes follicular growth and ovulation, and maintains gonadotropin hormones [45]. Cumulus granulosa cells produce plenty of IGF1 [46]. Overexpression of CD86, a macrophage molecular marker, contributes to POI [47]. Moreover, lncRNA may modulate signaling pathways that regulate cell adhesion, decrease apoptosis, and limit follicular transition and ovarian aging, which may explain how TAM protects the ovaries [14].

This study found that TAM increased serum NO and IL-10 levels more than CP-treated rats, indicating antioxidant and anti-inflammatory properties. CP-induced POI significantly downregulated IL-4 and IL-10 compared to a previous study [48]. Oxidative stress lowers NO, inflammation, and apoptosis, making CP-induced POI hazardous [49]. TAM also increased NO levels 24 h after administration [50]. TAM reduces oxidative damage, protecting granulosa cells, the developing oocyte, and follicular apoptosis. TAM can protect the ovary from immune-mediated damage and promote follicle growth by reducing inflammation.

Compared to the CP-treated rat, TAM therapy and pretreatment increased MFN-1 and DRP-1 levels, improving mitochondrial dynamics. MFN-1 and DRP-1, the main mitochondrial dynamics regulators, regulate oxidative metabolism, cell cycle regulation, and mitochondrial axonal transport [51]. Research has shown that mitochondrial dynamics affect female fertility. Small interfering RNA-induced inhibition of the mitochondrial fusion gene MFN-2 in immature oocytes reduces maturation, while oocyte-specific deletion of the mitochondrial fission factor DRP-1 impairs follicular maturation promotes female sterility [52, 53]. Additionally, MFN-1 is essential for female fertility and ovarian follicular reserve [54]. Maintaining mitochondrial dynamics with TAM can improve oocyte quality and competence, reduce oxidative stress, improve granulosa cell function, and delay ovarian aging.

The study found that TAM raised serum AMH. Medication increased primordial, primary, and atretic follicles, especially in the pTAM + CP group. Plasma levels of AMH are a good predictor of ovarian reserve because they reflect the pool of resting primordial follicles, which proliferate non-cyclically [55]. TAM preserves the ovary by preventing ovarian follicle transition [14]. TAM increased the production of inhibin α and AMH, which are involved in signaling pathways leading to primordial follicle activation or arrest [13]. TAM may have prevented primordial follicle depletion and increased early-stage follicle survival by increasing the biomarker for ovarian reserve. Our follicle count explorations may support this.

Rat ovary H&E, PAS, and Masson trichrome staining. It seems that ovarian follicles were ordered again. The pTAM-CP-pretreated rats improve more than the sTAM-CP group. Histology focuses on TAM-protective changes. Slices of ovarian follicles exhibited many cortical developing and a few damaged ones after H&E staining. The ovarian cortex of degenerated follicles had a single layer of stromal cells around the core main oocytes. The cortical primordial follicles and two multilaminar primary follicles with primary oocytes and zona pellucida membranes were close. Large secondary follicles with antral gaps inside granulosa cells and zona pellucida membrane-wrapped primordial oocytes were seen. Ovarian medulla blood channel dilatation and hyalinization were lower. PAS staining shows intact, thick zones around zones with minimal affection as a result of follicle zona pellucida changes. Sections stained with Masson trichrome showed a tiny collagen fiber area around follicles, suggesting minor TAM pretreatment fibrosis.

This supports the notion that TAM protects by raising HO-1 immunological marker expression in immunostained granulosa cells and lowering CD86 immunostained macrophage cells, especially in rats pretreated with TAM. More primordial, primary, and atretic follicles were related with more HO-1 immunostaining. TAM protects fertility and follicular loss following chemotherapy or irradiation by increasing AMH and local IGF-1 levels to counteract oxidative stress-induced apoptosis [11]. As previously reported, CP-treated ovarian tissue showed numerous atretic follicles and severe medulla degeneration and edema. Atretic follicles magnified showed granulosa cell shrinkage and nuclear pyknosis. The toxic effects of CP are caused by its active metabolites, which bind to DNA, disrupt DNA synthesis, and generate reactive oxygen species by conjugating glutathione, compromising the ovary’s antioxidant defense system and initiating an oxidative stress-induced apoptotic pathway [56].

Apoptosis of granulosa cells, thinning and separation of the zona pellucida membrane, loss or poorly defined oocytes, excessive collagen fiber accumulation in the ovarian stromal tissue, and follicular reserve depletion have been reported in ovarian growing follicles [57, 58]. According to several studies, chemotherapy-treated ovaries developed cortical stromal blood vessel fibrosis, thickness, and hyalinization. These data suggest chemotherapy-induced ovarian stroma and vascularization alterations may cause follicle loss [59–61]. To expedite endothelium restoration, TAM activates several cellular and molecular pathways, mimicking estrogen’s vasculoprotective effects [62]. TAM also decreased apoptosis and rescued the rat ovary from CP-induced follicular reserve depletion [63].

In the current study, tamoxifen increased the positive immunostaining reaction of HO-1 in granulosa cells, an indicator of oxidative stress, and CD86 in macrophage cells, a proinflammatory marker. This benefit may be due to TAM’s anti-apoptotic action, notably in pTAM-CP. Ferroptosis, a planned cell death that accumulates iron and peroxidizes lipids, also affects female reproduction. It impacts granulosa cell proliferation, oocyte development, ovarian reserve function, embryonic development, and placental oxidative stress [64]. HO-1 and glutathione peroxidase expression decreases, oxidative damage increases, and ferroptosis occurs [65]. TAM also affects macrophages, which fight infections first. TAM targets macrophage lipid mediators and signal pathways without the estrogen receptor, according to substantial evidence. These cells can adjust phenotypically and boost inflammatory response without dying [64].

After follicular rescue by TAM, targeted mRNA analysis revealed reduced expression of several inflammation-related genes. Genes involved in lipoxygenase and prostaglandin synthesis, signaling, cytokine binding, apoptosis, tissue remodeling, and vasodilation. These data support the idea that TAM prevents CP-mediated ovarian toxicity by directly preventing follicular loss [66]. In CP-induced POI, both pretreated and treated rats with TAM showed enhanced IGF-1R mRNA expression, while pretreated rats had considerably higher levels. Previous study has shown that TAM modulates the IGF-1/IGF-1R system in diverse tissues and directly affects IGF-1 secretion [11]. Contrary to previous research, CP may impair IGF-1-producing cells [67, 68]. TAM may also suppress lncRNAs and genes involved in ovarian steroidogenesis in rat ovaries, according to a recent study. TAM altered genes related to primordial follicle activation or arrest [13].

IGF-1 signaling by TAM boosts follicle numbers and decreases CP-induced apoptosis in rats in vitro [66]. Documentation shows that TAM injection increases interstitial ovarian cell IGF-1 and IGF-1R [44]. TAM also prevented follicle loss by modulating IGF-1/IGF-1R’s antioxidant and cytoprotective effects [11]. TAM may promote ovarian hyperstimulation, estrogen production, fertility, and ovulation via enhancing the IGF system.

In conclusion, TAM, particularly with pretreatment application, exerts its effects through several dependent pathways: firstly, an antioxidant pathway that enhances serum NO levels and increases HO-1 immunopositive cells; secondly, it promotes mitochondrial dynamic balance via the rebalancing of MFN-1 and DRP-1; thirdly, it activates an anti-inflammatory pathway by modulating IL-10, CD86, and the IGF system. To fully understand how TAMs affect human ovarian tissues in vivo, future prospective, concurrent translational studies are required.

## Data Availability

The datasets generated during and/or analyzed during the current study are available from the corresponding author on reasonable request.

## Abbreviations

AMH: Anti-Müllerian hormone
CD86: Cluster of differentiation 86
CP: Cyclophosphamide
DRP-1: Dynamin-related Protein
GF: Graffian follicles
HO-1: Heme Oxygenase 1
IGF-1: Insulin-like growth factor 1
IGF-1R: Insulin-like growth factor1 receptor
IL-10: Interleukin-10
MFN-1: Mitofusin 1
NO: Nitric oxide
POI: Premature ovarian insufficiency
SF: Secondary follicle
TAM: Tamoxifen
